# Chronic Antibody-Mediated Rejection and Plasma Cell ER Stress: Opportunities and Challenges with Calcineurin Inhibitors

**DOI:** 10.3390/ijms26062711

**Published:** 2025-03-18

**Authors:** Ching-Yi Tsai, Chih-Yuan Lee, Jia-Huang Chen, Chih-Kang Chiang

**Affiliations:** 1 Graduate Institute of Toxicology, College of Medicine, National Taiwan University, Taipei 100233, Taiwan; ci65725@gmail.com (C.-Y.T.); f04447010@ntu.edu.tw (J.-H.C.); 2 Department of Medical Research, National Taiwan University Hospital, Taipei 100225, Taiwan; 3 Department of Surgery, National Taiwan University Hospital, Taipei 100225, Taiwan; gs2119@gmail.com; 4 Organ Transplant Center, National Taiwan University Hospital, Taipei 100225, Taiwan; 5 Department of Integrated Diagnostics & Therapeutics, National Taiwan University Hospital, Taipei 100225, Taiwan

**Keywords:** chronic alloantibody-mediated rejection, calcineurin inhibitors, ER stress

## Abstract

Chronic alloantibody-mediated rejection (cAMR) remains a major challenge in transplant immunology, with no FDA-approved targeted therapies currently available. Despite advancements in cellular immunosuppression, effective strategies to mitigate alloantibody-mediated rejection are still lacking. This review provides a comprehensive overview of transplant rejection with a particular focus on the pathophysiology and therapeutic landscape of cAMR. We highlight the role of plasma cell-driven alloantibody production and its susceptibility to endoplasmic reticulum (ER) stress, a pathway with potential for therapeutic intervention. Special attention is given to calcineurin inhibitors (CNIs), which, beyond their well-established T-cell inhibitory effects, exhibit differential impacts on ER stress and plasma cell viability. By delineating the mechanistic differences between cyclosporine and tacrolimus in regulating ER stress responses, we propose potential therapeutic implications for optimizing cAMR management. This review underscores the need for innovative strategies targeting plasma cell biology to improve long-term transplant outcomes.

## 1. Introduction

Kidney transplantation represents one of the therapeutic options for patients with advanced chronic kidney disease or end-stage kidney disease (ESKD). More than 20 years of transplant data from the United States Scientific Registry of Transplant Recipients (SRTR) show that the one-year graft survival rate for kidney transplant recipients under immunosuppressive therapy has reached 95%. Approximately 50% of transplanted kidneys fail within 8 to 11 years [1]. Antibody-mediated rejection is the leading cause of immune-related allograft failure after renal transplantation. However, the U.S. Food and Drug Administration (FDA) has not yet approved any specific drugs for either acute or chronic AMR (cAMR), and no standardized treatment guidelines have been established [2,3]. Therefore, a comprehensive understanding of the pathophysiology and mechanism of AMR is critical for optimizing its management.

Endoplasmic reticulum (ER) stress occurs in response to the accumulation of unfolded proteins within the ER, potentially triggering cell apoptosis [4,5]. Plasma cells, the major producers of alloantibodies, are highly resistant to immunosuppressants but are vulnerable to ER stress [6]. Due to their high antibody secretion rates, plasma cells require ER expansion to meet this demand, making them particularly susceptible to ER stress. Herein, we summarize the differential ER stress responses induced by calcineurin inhibitors and their impact on plasma cells, which may inform the development of novel therapeutic strategies for antibody-mediated rejection.

## 2. Damage of Donor-Specific Antibodies in Organ Transplantation

### 2.1. Antibody-Mediated Rejection

After renal transplantation, AMR occurs when the donor-specific antibodies (DSAs) target the transplanted organ, leading to immune activation and subsequent graft damage. There are several phenotypes of AMR after transplantation that are determined by the characteristics of the DSAs, such as antigen classes, IgG subclasses, and their capacity for complement fixation. AMR in kidney transplantation was initially defined as a distinct clinicopathological entity in the 1997 International Banff Classification of renal allograft rejection, and it has since undergone regular updates to refine its diagnostic criteria [7]. While the definition of acute AMR was well established in the late 1990s and early 2000s, the understanding of chronic AMR remained ambiguous. cAMR is a progressive disease that typically develops months to years after organ transplantation when DSAs emerge and target the transplanted organ. Several pathological features of cAMR, such as arterial fibrinoid necrosis and microangiopathy, are associated with circulating donor-specific anti-donor human leukocyte antigen (HLA) antibodies. These antibodies can activate the complement system and release chemoattractants, leading to the recruitment of inflammatory cells in the peritubular capillaries [8]. Plasma cells play a key role in the adaptive immune response by secreting antibodies. Therefore, a deeper understanding of plasma cell biology may help in developing strategies to counteract AMR after transplantation.

### 2.2. Donor-Specific Antibodies and AMR

**Figure 1:**
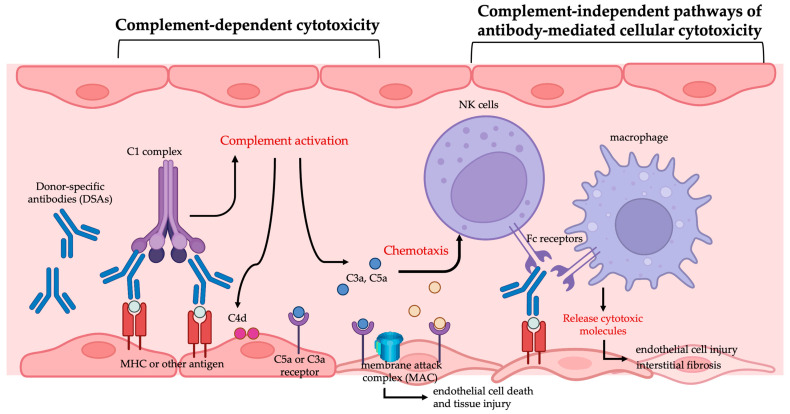
The mechanisms of donor-specific antibody-mediated endothelial injury in allografts. The mechanisms of injury involve complement activation and inflammation, which induce endothelial cell injury or death.

DSAs are associated with a higher risk of AMR and are recognized as an established biomarker for predicting AMR. Recent reports indicate that donor-specific antibodies were detected in 51% of patients with renal graft failure compared with only 2% in the control group with stable allografts. Additionally, in 60% of the patients with graft failure, DSAs were present before the graft failure occurred [9]. Terasaki et al. reported a prospective trial to test anti-HLA antibodies in the serum from 1329 patients with functioning renal grafts in the 13th International Histocompatibility Workshop, and they followed the patient for 4 years. They found that patients with detectable anti-donor HLA antibodies in their serum had lower graft survival rates [10]. This analysis confirms the association between DSAs and AMR. Despite immunosuppressive therapy, approximately 20% of the transplant recipients developed de novo DSAs [10]. The pathogenesis of DSA-induced AMR involves both complement-dependent cytotoxicity and complement-independent pathways such as antibody-mediated cellular cytotoxicity, as illustrated in Figure 1. The main target of DSAs is the vascular endothelium of the graft. When DSAs bind to antigens on the surface of endothelial cells in the graft, such as HLA molecules, they activate the complement system. Complement activation generates cleavage products like C3a and C5a, which possess chemotactic properties that attract more immune cells to the graft site. This activation of the complement system ultimately leads to the formation of the membrane attack complex, which directly disrupts the cell membrane, causing endothelial cell death and tissue injury [7]. In addition, to complement this activation, the Fc regions of antibodies can bind to Fc receptors (e.g., FcγR) on the immune cells, triggering antibody-dependent cellular cytotoxicity (ADCC). This process activates effector cells such as macrophages and natural killer (NK) cells, which release cytotoxic molecules like perforin and granzymes, destroying the antibody-targeted cells [11]. The cytotoxicity events lead to chronic graft vasculature injury, characterized by endothelial cell injury, transplant glomerulopathy (in kidney transplants), and interstitial fibrosis [12,13]. Unlike AMR, cAMR has a more gradual onset and a persistent course, which ultimately leads to progressive graft dysfunction and failure. Acute AMR occurs when the allograft is rapidly exposed to high levels of circulating DSAs, leading to complement activation, thrombosis, and leukocyte recruitment. In contrast, cAMR occurs due to prolonged exposure of donor endothelial cells to increasing levels of DSAs, leading to gradual cellular injury and inflammation. During this process, the graft may also undergo accommodation and repair mechanisms [14].

### 2.3. Diagnosis of AMR

One of the significant advancements in the pathology of kidney and other organ transplantation is the development of the Banff Classification. First developed in 1991, the Banff Classification system was designed to provide a standardized framework for transplant pathology diagnosis, allowing pathologists and clinicians worldwide to adopt a consistent approach to diagnose organ transplant rejection [15]. The Banff Classification has undergone multiple revisions and improvements. The 1997 Banff Conference further refined the diagnostic criteria for AMR, introducing its pathological and clinical features for the first time. Notably, C4d deposition was identified as a key marker of AMR [16]. As chronic rejection gained more attention, the Banff Classification system formally incorporated it at the 2005 conference [17]. At that time, chronic rejection was primarily characterized by transplant glomerulopathy and vascular fibrosis, prompting comprehensive research into its diagnosis, association with chronic lesions, and impact on long-term graft survival. The most recent revision, in 2019, further refined the definition of AMR, providing more detailed classifications for acute AMR (active AMR) and chronic active AMR (caAMR). Additionally, the revision emphasized the importance of complement activation and the persistent presence of antibodies in chronic rejection [18,19]. The pathological analysis of AMR involves detecting DSAs in the serum, identifying pathological evidence of acute microvascular inflammation such as increased glomerulitis or peritubular capillaritis scores, and assessing C4d complement deposition or other immune-mediated injury markers. A rapid decline in graft function further supports the diagnosis. According to the latest updates from the Banff Classification (2022), the guidelines for scoring lesions in renal allograft pathology consist of various components, each evaluating specific histopathological changes [20]. The detailed scoring criteria, including specific categories for lesion evaluation, are summarized in Table 1. C4d staining of allograft tissue is essential for classification assessment and is primarily performed using immunohistochemistry (IHC) on fresh-frozen or paraffin-embedded tissues [16,20]. According to the Sensitization in Transplantation: Assessment of Risk Working Group Meeting Report, testing for DSAs, including anti-HLA or other specificities, is recommended [21]. The diagnosis of AMR relies on the fulfillment of three key criteria: (1) histological evidence of AMR activity or chronicity, (2) confirmation of antibody interactions with the donor’s endothelium, and (3) serological evidence of DSAs. These criteria provide a comprehensive framework for accurately diagnosing and managing AMR in transplant recipients. [22,23]. For cAMR, the diagnostic criteria include the persistent presence of DSAs in the serum; chronic pathological damage such as transplant glomerulopathy, arteriosclerosis, and fibrosis, with or without C4d deposition; and a gradual decline in graft function accompanied by signs of chronic kidney damage [24,25]. As more data are collected and analyzed, the Banff Classification continues to evolve, providing a comprehensive assessment framework that enhances diagnostic precision and predictive insights for improved patient management.

### 2.4. Treatment Strategies for cAMR

Currently, treatment options for cAMR are limited, and research is ongoing to develop more effective therapies. Typically, the treatment involves a combination of therapies to reduce the production of DSAs and minimize damage to the transplanted organ. Several therapeutic strategies have been explored for the management of cAMR, as summarized in Table 2. These approaches target different aspects of the immune response and vary in efficacy, with some still under investigation.

#### 2.4.1. Plasmapheresis

In ABO-incompatible kidney transplantation, the recipient naturally possesses anti-A or anti-B antibodies that target the donor organ’s blood group antigens. These antibodies can trigger hyperacute rejection, leading to graft loss. To reduce this risk, plasmapheresis is commonly performed before transplantation to reduce antibody levels [26]. Plasmapheresis is a non-selective apheresis technique that separates plasma from whole blood using either centrifugation or filtration. The extracted plasma is then discarded and replaced with Ringer’s solution, albumin, and/or fresh-frozen plasma (FFP). To minimize plasma wastage, researchers have developed adsorption systems that specifically target IgG and IgM antibody subclasses, including HLA and anti-A/B antibodies. [27,28]. Through selectively removing DSAs from circulation, plasmapheresis serves as a crucial role in desensitization protocols and AMR management [3].

#### 2.4.2. Intravenous Immunoglobulin (IVIG)

IVIG is a blood-derived product primarily composed of immunoglobulin G (IgG) obtained from the pooled plasma of healthy human donors. It is widely utilized in the treatment of various inflammatory and autoimmune diseases. The immunomodulatory mechanisms of IVIG are complex, involving both IgG Fab-mediated antigen binding and Fc-mediated interactions with various soluble proteins and cell-surface receptors. Additionally, IVIG can inhibit the complement activation cascade, modulate dendritic cells to suppress cytokine production, and mitigate the autoimmune responses of T cells [29]. Moreover, studies have demonstrated that IVIG treatment leads to functional silencing of B cells, characterized by diminished intracellular calcium signaling, impaired B-cell receptor (BCR) clustering, and downregulation of coreceptor expression [30]. IVIG is commonly used in combination with plasmapheresis for desensitization therapy and the treatment of early AMR in transplant patients. For cAMR, several retrospective and prospective studies have investigated the efficacy of IVIG combined with plasmapheresis and rituximab. However, findings suggest that this combination is ineffective in patients with DSAs [31,32,33].

#### 2.4.3. Rituximab

Rituximab, a CD20-targeting monoclonal antibody, has been examined as a treatment for antibody-mediated rejection (AMR) in renal transplantation. In acute AMR, some studies suggest that multiple doses may improve graft survival and function. However, randomized controlled trials (RCTs) have not demonstrated a clear advantage over standard therapies such as plasmapheresis and IVIG. Its safety profile is generally comparable to alternative treatments. In cAMR, rituximab has shown inconsistent or unfavorable outcomes, likely due to irreversible tissue damage, with some reports indicating an increased risk of infection. While it may serve as a rescue therapy in severe acute AMR, its effectiveness in cAMR remains uncertain. Further, well-designed RCTs are required to better define its clinical role and long-term safety [34].

#### 2.4.4. Proteasome Inhibitors

Proteasome inhibitors such as bortezomib and carfilzomib have been explored as potential treatments for cAMR due to their ability to selectively target the plasma cells responsible for antibody production [35,36]. Although promising, these agents are still under investigation and are associated with adverse effects, including peripheral neuropathy and gastrointestinal complications [37]. In some studies, bortezomib has been used in combination with plasmapheresis or intravenous immunoglobulin (IVIG) to further reduce circulating DSAs and enhance treatment outcomes [38].

#### 2.4.5. Tocilizumab

Tocilizumab, an interleukin-6 (IL-6) receptor antagonist, has been utilized in various inflammatory diseases and is gaining attention in transplantation. Given the role of IL-6 in B-cell-mediated antibody production and inflammatory responses, IL-6 inhibition represents a potential therapeutic avenue. Studies have indicated that tocilizumab treatment in transplant recipients correlates with reduced antibody levels and improved graft function [2,39].

#### 2.4.6. Eculizumab

Eculizumab, a monoclonal antibody that inhibits complement component C5, has been employed to mitigate complement-mediated injury in AMR and atypical hemolytic uremic syndrome. Although its role in preventing cAMR is still being explored, eculizumab represents a promising future therapeutic option in transplant immunology [40,41].

## 3. Targeting Plasma Cells with Conventional Immunosuppressive Drugs: A Strategy to Mitigate Antibody-Mediated Rejection

Traditionally, managing immune responses after organ transplantation has focused on depleting T cells or suppressing their activity. However, as acute graft rejection has become more manageable, cAMR is now recognized as an underestimated factor contributing to the chronic deterioration of many allografts [42]. Plasma cells produce DSAs and other antibodies that lead to graft damage. Therefore, the treatment of cAMR involves not only the removal of circulating antibodies but also the suppression of antibody production by plasma cells [43].

### 3.1. The Function of the Endoplasmic Reticulum in Cells

The ER is an essential organelle in cells. Based on its structure and function, the ER can be divided into the rough endoplasmic reticulum (RER) and the smooth endoplasmic reticulum (SER). The RER has ribosomes attached to its surface and is involved in protein synthesis. The proteins generated through mRNA translation are folded and modified in the lumen of the RER. The ER also controls protein quality, ensuring that these proteins are properly folded. Misfolded proteins are identified and degraded, while properly folded proteins are transported to the Golgi apparatus, lysosomes, or the cell membrane. The SER lacks ribosomes and is primarily responsible for the synthesis of lipids, such as phospholipids, cholesterol, and steroid hormones. The SER is especially important in liver cells due to its role in the detoxification metabolism [44]. Additionally, the ER stores calcium ions within cells and releases them during signal transduction processes, regulating functions such as muscle contraction [45].

#### 3.1.1. Response to Stress in the Endoplasmic Reticulum

**Figure 2:**
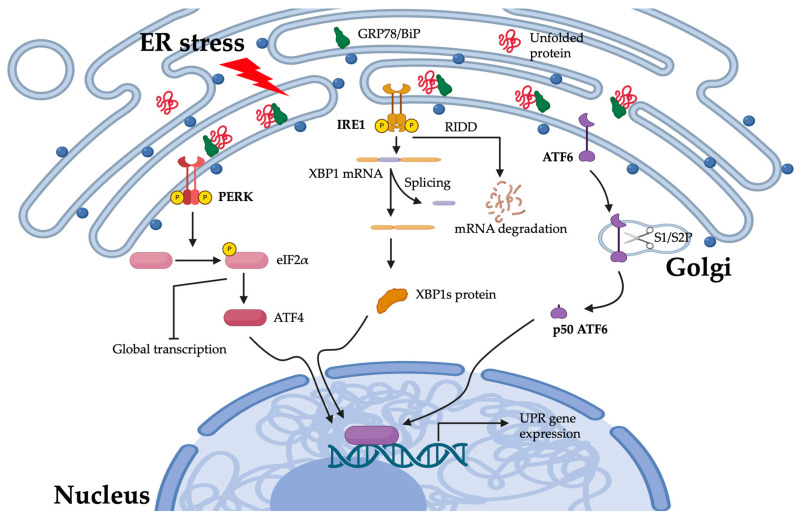
The mechanisms of UPR in the ER. Upon the accumulation of unfolded or misfolded proteins in the ER lumen, GRP78/BiP disassociates from IRE1, PERK, and ATF6, facilitating their activation and initiating the unfolded protein response. The PERK phosphorylates eIF2α, leading to the attenuation of translation while permitting the selective translation of ATF4. The endoribonuclease activity of IRE1 catalyzes the unconventional splicing of XBP1 mRNA, resulting in the formation of XBP1s mRNA, which encodes the active transcription factor XBP1s. Concurrently, in parallel, ATF6 translocates to the Golgi apparatus upon ER stress, where it undergoes site-1 (S1P) and site-2 (S2P) protease-mediated cleavage. This processing releases its cytosolic N-terminal domain (p50 ATF6), which functions as an active transcription factor. These pathways induce the expression of genes that enhance protein folding, quality control, and degradation, ultimately promoting cellular adaptation and restoring ER homeostasis. The image was created with Biorender, https://biorender.com.

Various intracellular and extracellular stressors, such as metabolic abnormalities [46], environmental toxins [47], or genetic factors [48], can lead to the accumulation of unfolded or misfolded proteins. Excessive protein misfolding in the ER disrupts protein-folding homeostasis, leading to ER stress. In response, the unfolded protein response (UPR) is activated as a cellular adaptive mechanism to detect and mitigate this imbalance. The UPR enhances the protein-folding capacity in the ER and promotes the degradation of misfolded proteins, thereby restoring ER function and maintaining proteostasis [49,50]. As illustrated in Figure 2, the UPR is mediated by three principal signaling pathways—protein kinase RNA-like ER kinase (PERK), inositol-requiring enzyme 1 (IRE1), and activating transcription factor 6 (ATF6)—which play essential roles in preserving cellular homeostasis under ER stress conditions.

#### 3.1.2. The PERK Pathway

PERK mediates the phosphorylation of eukaryotic initiation factor 2 alpha (eIF2α), resulting in a widespread attenuation of protein synthesis. This translational attenuation reduces the influx of newly synthesized proteins into the ER, thereby alleviating the protein-folding burden and allowing the cell to restore ER homeostasis. However, during prolonged ER stress, sustained eIF2α phosphorylation upregulates the transcription of activating transcription factor 4 (ATF4), which has been implicated in the regulation of autophagy. Additionally, ATF4 plays a pivotal role in apoptosis by promoting the expression of pro-apoptotic factors such as C/EBP homologous protein [51] and Noxa [52].

#### 3.1.3. The IRE1 Pathway

The IRE1 pathway is another critical arm of the UPR. Upon ER stress, IRE1 undergoes autophosphorylation, leading to the unconventional splicing of XBP1 mRNA, producing a potent transcription factor, XBP1 splicing (XBP1s), that upregulates the genes responsible for protein folding, ER-associated degradation (ERAD), and lipid synthesis. IRE1 also engages in regulated IRE1-dependent decay (RIDD) of mRNAs, further reducing the protein load in the ER [53].

#### 3.1.4. The ATF6 Pathway

ATF6, another transmembrane protein localized to the ER, plays a critical role in the UPR. Upon the onset of ER stress, ATF6 translocates to the Golgi apparatus, where it undergoes site-specific proteolytic cleavage to release its cytosolic domain. The activated cytosolic fragment of ATF6 subsequently translocates to the nucleus, where it functions as a transcription factor. In this capacity, ATF6 upregulates the expression of genes encoding ER chaperones such as BiP/GRP78 as well as components of the ERAD pathway, thereby enhancing the protein-folding capacity of the ER and the degradation processes to alleviate proteotoxic stress [54].

When the UPR successfully alleviates the accumulation of unfolded or misfolded proteins, the UPR signaling is attenuated, and cellular protein-folding homeostasis is restored. Conversely, if the imbalance persists and ER stress remains unresolved, apoptotic pathways are activated, ultimately leading to cell death.

### 3.2. The ER in Plasma Cells Is Closely Related to Antibody Production and Survival

Plasma cells are specialized antibody-producing cells derived from B cells. The differentiation of plasma cells is a complex process initiated by the activation of naive B cells upon encountering specific antigens with the assistance of T helper cells. During this differentiation, specific markers are expressed at various stages. Initially, naive B cells express markers such as CD19 and CD20. As they transition to plasmablasts, the expression of these markers diminishes, and CD138 (syndecan-1) becomes a prominent marker of the mature plasma cells [55]. As the center for protein synthesis, folding, and transport, the ER is crucial for antibody production in plasma cells. ER stress and the UPR are fundamental mechanisms for regulating protein balance in plasma cells.

#### 3.2.1. ER-Associated Molecular Regulation and Its Impact on the Antibody Production of Plasma Cells

XBP1 is significantly upregulated during plasma-cell differentiation, promoting the expansion of the ER and enhancing antibody synthesis efficiency [56]. The researchers employed gene expression profiling to examine the terminal differentiation of B cells derived from Blimp-1- and XBP1-deficient mice, aiming to elucidate the roles of these transcription factors in plasma cell development. The study demonstrated that Blimp-1 plays a pivotal role in the differentiation process of B cells into plasma cells by regulating the expression of XBP1s and ATF6 proteins, which are essential for ER expansion. Moreover, XBP1 promotes substantial ER expansion while also enhancing cell size, organelle biogenesis, and overall protein synthesis, underscoring its pivotal role in shaping the secretory cell phenotype. Plasma cells lacking Blimp-1 lose the ability to secrete antibodies [57,58]. Blimp-1 also modulates the activity of the mTOR kinase and influences plasma cell size. Therefore, B-cell differentiation and the formation of functional plasma cells capable of secreting protective antibodies require Blimp-1 [58]. It has been found that the inactivation of the transmembrane protein Jagunal homolog 1 (JAGN1), located on the ER membrane, leads to impaired plasma cell numbers in bone marrow, and JAGN1 deficiency is associated with reduced antibody production and secretion in plasma cells [59].

#### 3.2.2. Plasma Cell Survival and ER Stress: Molecule Mechanism and Regulation

**Figure 3:**
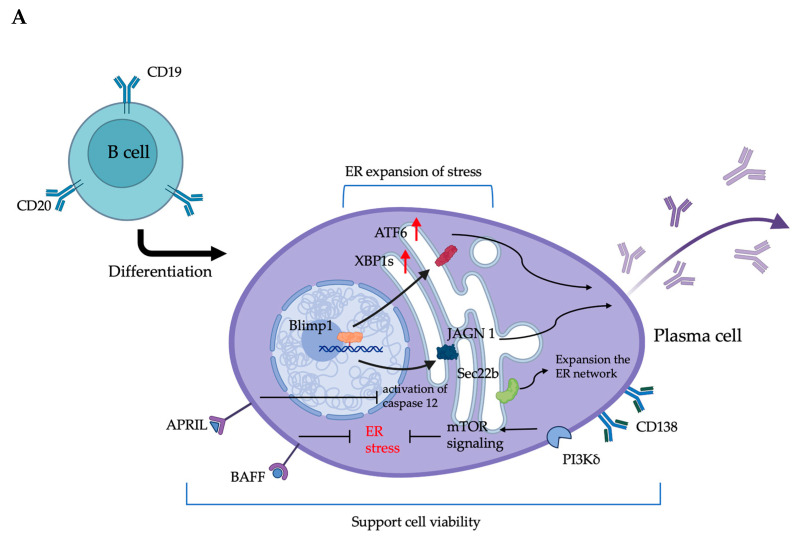

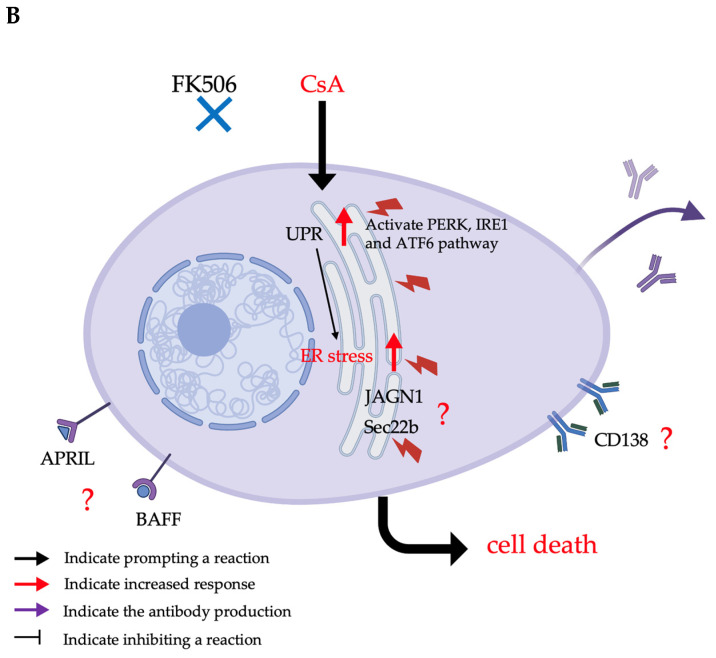
An overview of the molecules involved in antibody production and support of cell viability within the differentiation of plasma cells (**A**) and prediction of the effects of CNIs (**B**) in plasma cells. The image was created with Biorender, https://biorender.com.

Researchers have discovered that a proliferation-inducing ligand (APRIL) signaling through the nuclear factor kappa B (NF-κB) pathway in plasma cells inhibits the activation of caspase 12, a key initiator of ER stress-induced apoptosis [60]. Additionally, the B-cell activating factor from the TNF family (BAFF) has been shown to regulate autophagy, a process vital for maintaining protein quality control and cellular stress responses, thereby potentially modulating how plasma cells cope with ER stress [61]. APRIL and BAFF, secreted by stroma cells, are essential for maintaining plasma cell survival by binding to their receptors, such as BCMA (B-cell maturation antigen) and TACI (transmembrane activator and CAML interactor). These interactions promote the plasma cells’ longevity and support their ability to secrete antibodies. Studies have shown that these factors help plasma cells survive in specific niches, such as the bone marrow, by counteracting pro-apoptotic signals and supporting the UPR under conditions of stress [62]. Recent studies have indicated that CD138, expressed on the surface of plasma cells, not only functions as an adhesion molecule but also enhances APRIL signaling by binding to heparan sulfate chains on the cell surface, thereby promoting plasma cell survival [51,63]. Moreover, a study has shown that the soluble N-ethylmaleimide-sensitive factor attachment protein receptor (SNARE) Sec22b regulates the cell cycle, ER–Golgi protein trafficking, and mitochondrial function. While Sec22b deficiency does not affect plasma-cell differentiation, it impairs ER network expansion, reduces ER–mitochondrial membrane contact sites, and leads to excessive mitochondrial fusion. These disruptions ultimately compromise plasma cell adaptability and survival [64]. It has been reported that protein synthesis regulation is related to mTOR activity during the LPS-induced differentiation of B cells into plasma cells. However, the genetic ablation of the tuberous sclerosis complex (TSC), a negative regulator of mTOR, resulted in increased apoptosis of the developing plasma cells [65]. Furthermore, mTORC1 signaling could be enhanced by increased PI3Kδ activity, which promotes elevated ER stress and impaired autophagy, leading to increased apoptosis in plasma cells. This suggests that PI3Kδ activity may play a regulatory role in specific immune responses [66]. A summary of the survival mechanisms involved in plasma cells is depicted in Figure 3A. During the differentiation of plasma cells, Blimp-1 regulates the cells to support the ER response, which involves the upregulation of XBP1s and ATF6, ultimately facilitating the production of large quantities of antibodies. In addition, JAGN1 and Sec22b, which are localized on the ER membrane, are involved in regulating antibody production and maintaining the integrity of the ER network. Moreover, APRIL, BAFF, mTOR, and CD138 are critical in maintaining survival signals for plasma cells.

### 3.3. The Effects of Immunosuppressive Drugs, Particularly Calcineurin Inhibitors, on ER Stress

Calcineurin inhibitors (CNIs) are a class of immunosuppressive drugs that primarily inhibit calcineurin activity, thereby preventing T-cell activation. Clinically, the most commonly used calcineurin inhibitors (CNIs) after organ transplantation are cyclosporine (CsA) and tacrolimus (FK506).

#### 3.3.1. The Mechanism of Calcineurin Inhibitors

CsA is a lipophilic cyclic peptide isolated from the fungus *Hypocladium inflatum gams* and was discovered by Sandoz (now Novartis). Researchers have found that it suppresses immune responses, and it has been used in transplantation [67]. The mechanism of CsA involves blocking the expression of IL-2 genes in activated T cells, thereby inhibiting T-cell activation [68]. Further studies revealed that the CsA–cyclophilin complex inhibits the calcineurin-mediated dephosphorylation, which in turn prevents the activation of the nuclear factor of activated T-cell (NFAT) transcription factor, thus blocking the transcriptional activation of genes such as IL-2, IL-4, and CD40L in T cells [69]. CsA can also block antigen recognition-triggered JNK and p38 signaling pathways in T cells, affecting their survival [70,71]. Subsequently, in 1984, tacrolimus (also known as FK506) was isolated from the fermentation broth of *Streptomyces tsukubaensis* in Japan. This macrolide antibiotic also exhibits immunosuppressive effects in vitro [72]. In T cells, tacrolimus can rapidly and directly inhibit IL-2 gene transcription as well as other activation-related genes, such as IL-3, IL-4, TNF-α, IFN-γ, and c-myc [73]. Its mechanism of inhibiting T-cell activity involves binding to FK506-binding protein 12, which subsequently inhibits the transcriptional activity of the NFAT transcription factor [74]. Both CsA and FK506 are metabolized through the CYP3A enzyme system in the liver. However, FK506 undergoes more rapid metabolism, resulting in a shorter half-life compared with CsA. FK506 is more potent than CsA, allowing for lower required doses. Nevertheless, due to its higher potency, FK506 must be used with greater caution to prevent excessive immunosuppression and associated toxicity [75]. CsA and FK506 play a significant role in preventing rejection in organ transplant recipients. Although their mechanisms of action are similar, there are distinct differences between them [76].

#### 3.3.2. Side Effects of Calcineurin Inhibitors

Clinical and basic research has revealed that CNIs affect multiple systems, their side effects including nephrotoxicity, neurotoxicity, and hepatotoxicity. Previous studies on CNI toxicity indicated that CsA presents a higher risk of renal injury compared with FK506, as it is associated with chronic interstitial fibrosis, tubular atrophy, and endothelial cell damage in the kidneys [70,77]. Although FK506 can also cause nephrotoxicity, its mechanism tends to involve vasoconstriction and increased intracellular calcium influx, leading to acute kidney injury [78]. Regarding neurotoxicity, FK506 appears to have more pronounced effects, especially at higher doses. Common side effects include tremors, headaches, insomnia, and mental confusion. CNIs may induce neurotoxicity through several potential mechanisms, such as compromising the integrity of the blood–brain barrier, disrupting mitochondrial function, and altering neuronal electrophysiology [79,80,81]. In organ transplant patients, CsA-induced hypertension is common, and the rise in blood pressure may stem from increased oxidative stress and inflammation in the vascular wall, leading to vascular sclerosis and fibrosis. Activation of the renin–angiotensin–aldosterone system (RAAS) is another factor contributing to hypertension. Additionally, CsA significantly impacts lipid profiles, increasing the risk of hypercholesterolemia and atherosclerosis. Studies have observed that FK506 may cause hypertension due to increased oxidative stress and the activation of angiotensin II-dependent vasoconstriction pathways [82]. However, treatment with FK506 may significantly contribute to the development of hyperlipidemia in animal experiments [83], but the incidence of hyperlipidemia was lower compared with treatment with CsA [82,84]. Although CNIs pose toxicity risks, they remain the preferred choice for managing allograft rejection until more suitable alternatives are developed.

#### 3.3.3. The Potential cAMR Treatment of CsA Through the ER Stress Effect on Plasma Cells

In recent years, researchers have focused on the potential of developing or discovering drugs that inhibit plasma cell function for the treatment of cAMR. Looking back, it is important to understand the effects of CNIs on cellular ER function, because plasma cells are vulnerable to ER stress. Previous studies have shown that CsA exerts different effects on ER stress compared with FK506. Clinical observations have indicated that CsA is more nephrotoxic than FK506, leading researchers to investigate the mechanisms behind CsA-induced nephrotoxicity. It has been found that both CsA and high doses of FK506 induce the UPR, inhibiting NF-κB activation and thus reducing TNF-α response [85]. Another study demonstrated that endothelial cells exposed to a therapeutic concentration of 10 µM CsA trigger ER stress, resulting in alterations to the endothelial phenotypic changes (EPCs), a decrease in CD31 expression, and subsequent cell death. In contrast, FK506 at the same concentration does not alter the cellular morphology, even at levels 1000 times higher than the therapeutic concentration [86]. Researchers further investigated the UPR pathway triggered by CsA-induced ER stress. The results indicated that CsA activates AMP-activated protein kinase, GCN2, and PERK in human epithelial cells, leading to reduced protein synthesis via eIF2α phosphorylation. Furthermore, experiments using human embryonic kidney cells demonstrated that CsA induces ER stress via activation of the PERK and ATF6 pathways, ultimately resulting in cell death. Notably, these effects were absent under the FK506 treatment [87,88]. From previous research results, it has been speculated that CsA and FK506 exert significantly different effects on the ER of plasma cells. In the context of controlling or eliminating antibody secretion from plasma cells, the role of CsA in inducing UPR offers a direction for further investigation. Our recent experiments have demonstrated that CsA exerts cytotoxic effects on plasma cells, whereas FK506 exhibits minimal impact. These findings may provide valuable insights into selecting drug combinations for preventing both cellular rejection and antibody-mediated rejection. Figure 3B summarizes the potential effects of CNIs on the ER. The impact of CsA on plasma cells may be more significant than that of FK506. CsA is expected to induce stress in plasma cells, reducing antibody production and promoting cell death. Currently, it remains unproven whether CsA influences the factors APRIL, BAFF, sec22b, and CD138, which support the survival of plasma cells, as there are no existing studies addressing this question.

## 4. Conclusions

**Figure 4:**
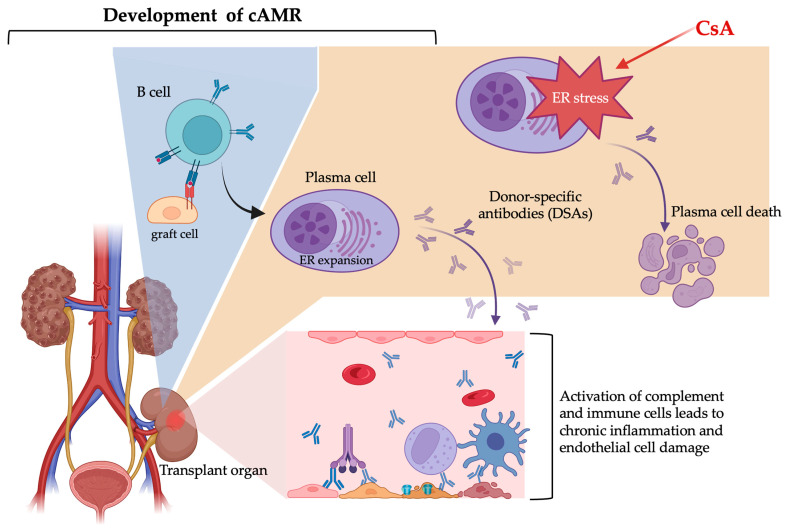
ER stress-induced plasma cell apoptosis as a therapeutic target in cAMR. cAMR is a leading cause of late allograft failure, driven by persistent DSA production from long-lived plasma cells. Plasma cells are highly susceptible to ER stress due to their intense secretory demands, and excessive ER stress can trigger apoptosis, reducing DSA levels. Targeting this issue, the immunosuppressive drug CsA could enhance ER stress, selectively promoting plasma cell death and thereby slowing cAMR progression. Exploring ER stress as a therapeutic mechanism offers novel strategies to control cAMR and improve long-term transplant outcomes. The image was created with with Biorender, https://biorender.com.

Plasma cells are capable of producing large quantities of antibodies within short timeframes. The high biosynthetic demands of plasma cells necessitate a highly functional ER. Any imbalance in protein synthesis, folding, or assembly or of the secretion of antibodies in plasma cells can lead to ER stress or even apoptosis. The cellular response to the substantial burden of secretory protein synthesis and ER stress helps to maintain homeostasis and prevents apoptosis. In recent years, research on ER stress in plasma cells has gained momentum as a potential therapeutic approach for treating antibody-mediated rejection. Understanding the molecular pathways involved in ER stress is uncovering an entirely new set of potential therapeutic strategies. This study investigates the differential effects of conventional CNIs on plasma cells. Based on previous research and immune response mechanisms, it can be inferred that CsA exhibits superior clinical efficacy in controlling cAMR compared with FK506. We summarize the key point of this paper in Figure 4. However, due to concerns regarding the higher risk of the adverse effects of CsA, future strategies may necessitate combination therapies to optimize their benefits. Effectively managing antibody-mediated rejection while minimizing drug toxicity requires more extensive future research.

## Figures and Tables

**Table 1:** The lesion scoring of the Banff Classification.

Banff Lesion Score	Pathological Indicator	Scoring Criteria
i	Interstitial Inflammation	i0—No inflammation or < 10% of unscarred cortical parenchyma. i1—Inflammation in 10-25% of unscarred cortical parenchyma. i2—Inflammation in 26-50% of unscarred cortical parenchyma. i3—Inflammation in more than 50% of unscarred cortical parenchyma.
t	Tubulitis	t0—No tubulitis. t1—1~4 mononuclear cells per tubular cross-section or per 10 tubular epithelial cells. t2—5~10 mononuclear cells per tubular cross-section or per 10 tubular epithelial cells. t3—>10 mononuclear cells per tubular cross-section or per 10 tubular epithelial cells.
v	Intimal Arteritis	v0—No arteritis. v1—Mild to moderate intimal arteritis in at least 1 arterial cross-section. v2—Severe intimal arteritis with at least 25% luminal area lost in at least 1 arterial cross-section. v3—Transmural arteritis and/or arterial fibrinoid change and medial smooth muscle necrosis with lymphocytic infiltrate in vessel.
g	Glomerulitis	g0—No glomerulitis. g1—Glomerulitis in <25% of glomeruli. g2—Segmental or global glomerulitis in about 25–75% of glomeruli. g3—Glomerulitis (mostly global) in >75% of glomeruli.
ptc	Peritubular Capillaritis	ptc0—Maximum number of leukocytes < 3. ptc1—At least 1 leukocyte cell in ≥10% of cortical PTCs with 3–4 leukocytes in the most severely involved PTC. ptc2—At least 1 leukocyte in ≥10% of cortical PTCs with 5–10 leukocytes in the most severely involved PTC. ptc3—At least 1 leukocyte in ≥10% of cortical PTCs with >10 leukocytes in the most severely involved PTC.
C4d	C4d Deposition	C4d0—No staining of PTC and medullary vasa recta (0%). C4d1—Minimal C4d staining (>0 but <10% of PTC and medullary vasa recta). C4d2—Focal C4d staining (10–50% of PTC and medullary vasa recta). C4d3—Diffuse C4d staining (>50% of PTC and medullary vasa recta).
ci	Interstitial Fibrosis	ci0—Interstitial fibrosis in up to 5% of cortical area. ci1—Interstitial fibrosis in 6 to 25% of cortical area (mild interstitial fibrosis). ci2—Interstitial fibrosis in 26 to 50% of cortical area (moderate interstitial fibrosis). ci3—Interstitial fibrosis in >50% of cortical area (severe interstitial fibrosis).
ct	Tubular Atrophy	ct0—No tubular atrophy (defined as tubules with a thickened basement membrane or a reduction of greater than 50% in tubular diameter). ct1—Tubular atrophy (see ct0) involving up to 25% of the area of cortical tubules. ct2—Tubular atrophy (see ct0) involving 26 to 50% of the area of cortical tubules. ct3—Tubular atrophy (see ct0) involving >50% of the area of cortical tubules.
cv	Vascular Fibrous Intimal Thickening	cv0—No chronic vascular changes. cv1—Vascular narrowing of up to 25% luminal area by fibrointimal thickening. cv2—Vascular narrowing of 26 to 50% luminal area by fibrointimal thickening. cv3—Vascular narrowing of more than 50% luminal area by fibrointimal thickening.
cg	Glomerular Basement Membrane Double Contours	cg0—No double contours of glomerular basement membrane (GBM). cg1a—No double contours by light microscopy (LM), but present in ≥3 capillaries by transmission (EM) with endothelial swelling or subendothelial widening. cg1b—Double contours in 1~25% of capillary loops in the most affected glomerulus by LM. cg2—Double contours in 26–50% of capillary loops in the most affected glomerulus. cg3—Double contours in > 50% of capillary loops in the most affected glomerulus.
mm	Mesangial Matrix Expansion	mm0—Mild increase in mesangial matrix. mm1—Moderate increase in up to 25% of non-sclerotic glomeruli. mm2—Moderate increase in 26–50% of non-sclerotic glomeruli. mm3—Moderate increase in >50% of non-sclerotic glomeruli.
ah	Arteriolar Hyalinosis	ah0—No hyaline thickening. ah1—Mild to moderate thickening in ≥1 arteriole. ah2—Moderate to severe thickening in >1 arteriole. ah3—Severe thickening in many arterioles.
aah	Hyaline Arteriolar Thickening	aah0—No typical lesions of calcineurin inhibitor-related arteriolopathy. aah1—Hyaline deposits in 1 arteriole, no circumferential involvement. aah2—Hyaline deposits in >1 arteriole, no circumferential involvement. aah3—Circumferential hyaline deposits in arterioles, regardless of number.
ti	Total Inflammation	ti0—No or minimal inflammation (<10%). ti1—10–25% of cortex inflamed. ti2—26~50% of cortex inflamed. ti3—>50% of cortex inflamed due to interstitial inflammation and tubulitis
i-IFTA	Inflammation in Areas of Interstitial Fibrosis and Tubular Atrophy	i-IFTA0—No inflammation or <10% of cortex with fibrosis and atrophy. i-IFTA1—Inflammation in 10~25% of cortex with fibrosis and atrophy. i-IFTA2—Inflammation in 26~50% of cortex with fibrosis and atrophy. i-IFTA3—Inflammation in >50% of cortex with fibrosis and atrophy.
t-IFTA	Tubulitis in Areas of Interstitial Fibrosis	t-IFTA0—No mononuclear cells or single focus of tubulitis. t-IFTA1—2+ foci with 1~4 mononuclear cells/tubule in the most affected focus. t-IFTA2—2+ foci with 5~10 mononuclear cells/tubule in the most affected focus. t-IFTA3—2+ foci with >10 mononuclear cells/tubule in the most affected focus.
pvi	Polyomavirus Load	pvi0—No positive nuclei in any tubules/ducts. pvi1—≤1% of all tubules/ducts. pvi2—>1% to ≤10% of all tubules/ducts. pvi3—>10% of all tubules/ducts.

**Table 2:** Summary of the drugs used for treating cAMR.

Treatment Strategy	Representative Drug/Method	Purpose	Effectiveness	Side Effects
Antibody Removal Therapy	Plasma exchange	Remove circulating antibodies, reduce antibody levels in the short term	Effective in the short term	Temporary effect, requires repeated procedures
	IVIG	Neutralize antibodies, inhibit complement response		
B-cell Inhibitors	Rituximab (anti-CD20 antibody)	Reduce B cells, lower antibody production	Effective for some patients	Increased risk of infection
Emerging Therapies	Bortezomib, carfilzomib	Suppress plasma cells, reduce antibody production	Effectively reduces inflammation and tissue damage	Increased infection risk, peripheral neuropathy, gastrointestinal discomfort
	Tocilizumab (anti-IL-6)	Inhibit IL-6 activation, reduce inflammatory response		Increased infection risk
Complement Inhibitor	Eculizumab	Inhibit complement activation, reduce immune response	Effective for reducing antibody-mediated rejection and atypical hemolytic uremic syndrome	Increased risk of infections

## Data Availability

Not applicable.

## Abbreviations

ER	Endoplasmic reticulum
ESKD	Kidney disease or end-stage kidney disease
SRTR	Scientific Registry of Transplant Recipients
AMR	Antibody-mediated rejection
cAMR	Chronic antibody-mediated rejection
FDA	U.S. Food and Drug Administration
HLA	Human leukocyte antigen A
DSAs	Donor-specific antibodies
Mac	Membrane attack complex
Adcc	Antibody-dependent cellular cytotoxicity
Nk	Natural killer
IVIG	Intravenous immunoglobulin
IL-6	Interleukin-6
RER	Rough endoplasmic reticulum
SER	Smooth endoplasmic reticulum
UPR	Unfolded protein response
PERK	Protein kinase RNA-like ER kinase
IRE1	Inositol-requiring enzyme 1
CHOP	C/EBP homologous protein
Noxa	Phorbol-12-myristate-13-acetate-induced protein 1; PMAIP1
ERAD	ER-associated degradation
RIDD	Regulated IRE1-dependent decay
BiP/GRP78	Binding immunoglobulin protein or 78 kDa glucose-regulated protein
XBP1	X-box-binding protein 1
XBP1s	XBP1 splicing protein
ATF6	Activating transcription factor 6
Blimp-1	B-lymphocyte-induced maturation protein 1
mTOR	Mammalian target of rapamycin
mTORC1	Mammalian target of rapamycin complex
PI3Kδ	Phosphatidylinositol 3-kinase delta isoform
Sec22b	SEC22 homolog B recombinant protein
APRIL	Proliferation-inducing ligand
NF-κB	Nuclear factor kappa B
BAFF	B-cell activating factor belonging to the TNF family
BCMA	B-cell maturation antigen
TACI	Transmembrane activator and CAML interactor
CNI	Calcineurin inhibitors
CsA	Cyclosporine A
NFAT	Nuclear factor of activated T cells
TNF-α	Tumor necrosis factor-alpha
CYP3A	Cytochrome P450, family 3, subfamily A
RAAS	Renin–angiotensin–aldosterone system
EPC	Endothelial phenotypic changes
GCN2	General control nonderepressible 2
